# *Eimeria sciurorum* (Apicomplexa, Coccidia) From the Calabrian Black Squirrel (*Sciurus meridionalis*): An Example of Lower Host Specificity of Eimerians

**DOI:** 10.3389/fvets.2020.00369

**Published:** 2020-07-28

**Authors:** Jana Kvicerova, Lada Hofmannova, Francesca Scognamiglio, Mario Santoro

**Affiliations:** ^1^Department of Parasitology, Faculty of Science, University of South Bohemia, České Budějovice, Czechia; ^2^Department of Pathology and Parasitology, Faculty of Veterinary Medicine, University of Veterinary and Pharmaceutical Sciences Brno, Brno, Czechia; ^3^Department of Animal Health, Istituto Zooprofilattico Sperimentale del Mezzogiorno, Portici, Italy; ^4^Department of Integrative Marine Ecology, Stazione Zoologica Anton Dohrn, Naples, Italy

**Keywords:** squirrels, coccidia, host specificity, endemic species, parasite dispersal, southern Italy

## Abstract

Host specificity plays one of the key roles in parasitism. It affects the evolution and diversification of both host and parasite, as well as it influences their geographical distribution, and epidemiological significance. For most of parasites, however, host specificity is unknown or misrepresented because it is difficult to be determined accurately. Here we provide the information about the lower host specificity of *Eimeria sciurorum* infecting squirrels, and its new host record for the Calabrian black squirrel *Sciurus meridionalis*, a southern Italian endemic species.

## Introduction

The host specificity of a parasite—the degree to which the parasite is adapted to its host/s, and the number of host species that can successfully be used by the parasite—is the result of a long-term adaptation process undergone by both the host and the parasite (1, 2). It has a multifaceted nature, cohering with historical events (phylogeny, host–parasite coevolution), ecological conditions, and geography. Parasites are usually highly adapted to the environment of the specific host/s; thus, their ability to survive in other, differing environments may be limited (3, 4).

Highly host-specific parasites are strongly and well-adapted to their host and its environment, which facilitates their sustainability and longevity. Nevertheless, if the host becomes extinct, the parasite is also likely to become extinct. On the other hand, parasites with low host specificity are capable of exploiting several different host species (or even host genera, families, orders), which increases their opportunities for dispersal and for adapting to new environments (5, 6). Host specificity also affects the genetic structure of the parasite species/populations. Multihost parasites exhibit lower genetic diversity/reduced population structures in comparison to parasites with narrow host spectra due to their greater ability to disperse, which, in turn, results in increased gene flow between the parasites infecting each different host (1, 6–8).

The spectrum of host species of the particular parasite (or its life stage) is one of its fundamental characteristics; however, it is unknown for most parasite species. Therefore, it is often under- or overestimated. It can be studied and measured via several approaches, each of which, however, has its limitations: (i) The experimental cross-transmission study represents the traditional method by which new host-parasite combinations are created and tested, and thus offer an excellent, straightforward option. However, it is limited by the availability of experimental hosts and suitable and viable parasite stages. (ii) The thorough sampling of hosts for parasites is a rational method; however, there is never certainty that all suitable localities and a sufficient number of host individuals have been sampled. (iii) The reconstruction of host and parasite phylogenies offers information on the historical events of host-parasite associations, and can be used to make predictions about the conditions for colonization of a new host or new host lineages; nevertheless, it is necessary to obtain the sequences of the organisms to be studied (3, 4, 9).

Here we focus on *Eimeria* (Coccidia: Eimeriorina), a group of apicomplexan parasites that are distributed worldwide, some of them being of health and economic importance. They are evolutionarily older organisms, generally considered to be strictly host-specific, especially members of the genus *Eimeria* [e.g., (2, 10, 11)]. However, this has turned out not to be the rule. Several studies have shown that some species (e.g., *Eimeria chinchillae*, eimerians parasitizing mice of the genus *Apodemus*, but also *Eimeria sciurorum*) exhibit rather low host specificity (12–14). This study provides another example of the lower host specificity of *E. sciurorum* that was revealed based on field studies, coproscopical examination, and phylogenetic analyses. We also report a new host record for this coccidium, the Calabrian black squirrel (*Sciurus meridionalis*), which is an endemic species occurring in southern Italy (Calabria and Basilicata regions). It is also the first species of coccidium reported from this host.

## Materials and Methods

### General Data

A carcass of an adult male (485 g in weight) Calabrian black squirrel was collected on July 27, 2018 from the road SS283 in Fagnano Castello (Cosenza province), Calabria region, Italy. The Calabrian black squirrel was a fresh roadkill with a good nutritional status. Because of the occasional collection, the carcass was frozen at −20°C until necropsy, which was performed 5 days later. A thorough necropsy was performed by conventional techniques. During the necropsy, heart, blood vessels, trachea, lungs, urinary bladder, liver, gall bladder, kidneys, esophagus, stomach, and intestine were examined for helminths. Organs and tissues were opened, and their surfaces were first examined visually and then under a dissecting microscope (Leica M165C). Fecal samples were obtained at necropsy from the intestine, and a centrifugation-flotation concentration method with modified Sheather's sugar solution (15) and a standard sedimentation method were used to detect oocysts and eggs of helminths. When oocysts of coccidia were observed, an aliquot of feces was preserved in 96% ethanol and sent to the Department of Pathological Morphology and Parasitology, Faculty of Veterinary Medicine, University of Veterinary and Pharmaceutical Sciences, Brno, Czech Republic. Before the molecular analysis, the presence of coccidian oocysts was confirmed by direct microscopic observation of a drop of the ethanol-fixed sample using light microscopy (Olympus BX53 microscope with Nomarski interference contrast).

### Molecular Analyses

Identification of the oocysts was followed by DNA amplification, sequencing, and phylogenetic analyses. DNA was extracted from a 200 μl suspension of the ethanol-fixed fecal sample containing oocysts, after centrifugation and desiccation of the yielded pellet using the commercial GeneAll Exgene^TM^ Stool DNA mini kit (Cambio, UK) according to the manufacturer's instructions. A partial small subunit of 18S rRNA and a mitochondrial gene for cytochrome c oxidase subunit I (COI) were amplified following the PCR protocols and PCR primers published by (16). PCRs were performed in a 25 μl volume containing 3 μl (1–10 ng) of total DNA, 12.5 μl of commercial premix PCRBIO Taq mix red (PCRBiosystems, UK), 1 μl (400 μM) of each primer, and 7.5 μl of PCR H_2_O. Each PCR was performed with a negative control containing PCR H_2_O instead of DNA. As positive controls, DNA of *E. caviae* oocysts from guinea pig feces and DNA of *E. lancasterensis* oocysts from *Sciurus carolinensis* feces were used. The PCR products were separated by electrophoresis in 1.5% agarose gel stained with GoodView (ECOLI, Slovakia). Amplicons of expected sizes were purified using ExoSAP-IT® PCR Product Cleanup (Affymetrix, USA). Sequencing of the purified PCR amplicons was carried out by the commercial company Macrogen, Inc. (Amsterdam, the Netherlands). PCRs and sequencing of the obtained PCR products were performed for each of the amplified gene multiple times to make sure that correct sequences without errors were obtained.

### Phylogenetic Analyses

Obtained sequences were verified by BLAST (https://blast.ncbi.nlm.nih.gov/Blast.cgi), assembled and edited using the SequenceScanner v.1.0 (Applied Biosystems) and DNASTAR v.5.05 program package (DNASTAR, Inc., Madison, Wisconsin, USA), and deposited in the GenBank database under accession numbers MN650661 (18S rDNA) and MN657229 (COI). Alignments were created and adjusted in the BioEdit v.7.0.5 program (17). The 18S rDNA sequences were aligned in the nucleotide mode; the COI sequences were aligned in the amino acid mode, then switched to the nucleotide mode, and used for the analyses. Phylogenetic analyses were reconstructed using two approaches, the Bayesian inference (BI) and maximum likelihood (ML), computed in MrBayes v.3.2.2 (18) and Phyml v.2.4.3 (19), respectively. The most suitable model of evolution was determined using the jModelTest (20, 21). ML was computed using the GTR+Γ+I evolutionary model and the non-parametric bootstrap analysis of 1,000 replicates. BI was performed with parameters corresponding to the selected model (GTR+Γ+I), mcmc run for 10 million generations, and the tree sampling every 100 generations; the trees were summarized after removing 25% burn-in. Final trees were visualized and exported using TreeView v.1.6.6 (22) and adjusted in the Adobe Illustrator CS5 v.15.0 (Adobe Systems, Inc.). Pairwise similarities were calculated in PAUP^*^ v.4.0b10 (23).

## Results

**Figure 1:**
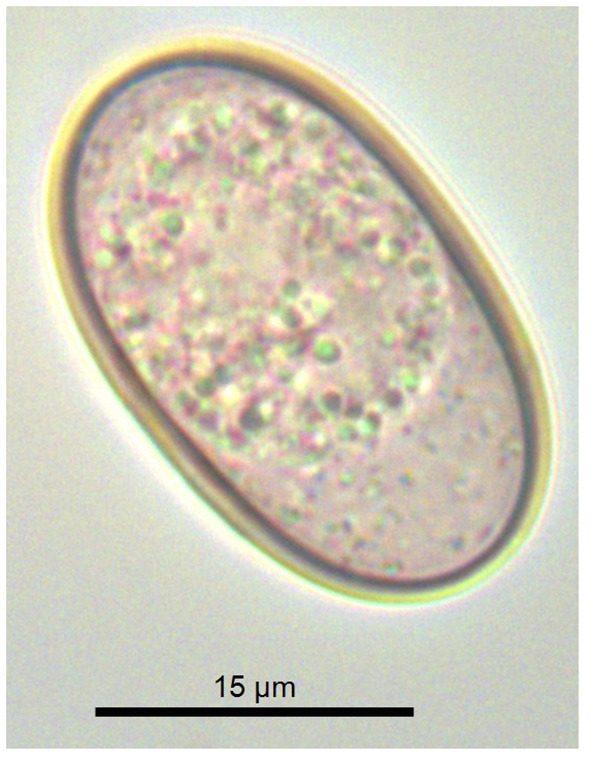
Unsporulated oocyst of *Eimeria sciurorum* from the feces of the Calabrian black squirrel (*Sciurus meridionalis*).

Post-mortem examination of the Calabrian black squirrel revealed multiple bone fractures, and hepatic and splenic laceration with severe hemoperitoneum. The parasitological examination revealed no helminths or their eggs. The coproscopical examination of the sample revealed only unsporulated coccidian oocysts, which were ellipsoidal to cylindrical (25–34 × 12–20 μm) with a smooth, bi-layered wall 1–2 μm thick, without a micropyle (Figure 1). Detailed morphological examination was not possible due to unsuccessful sporulation (the cadaver was initially frozen).

**Figure 2:**
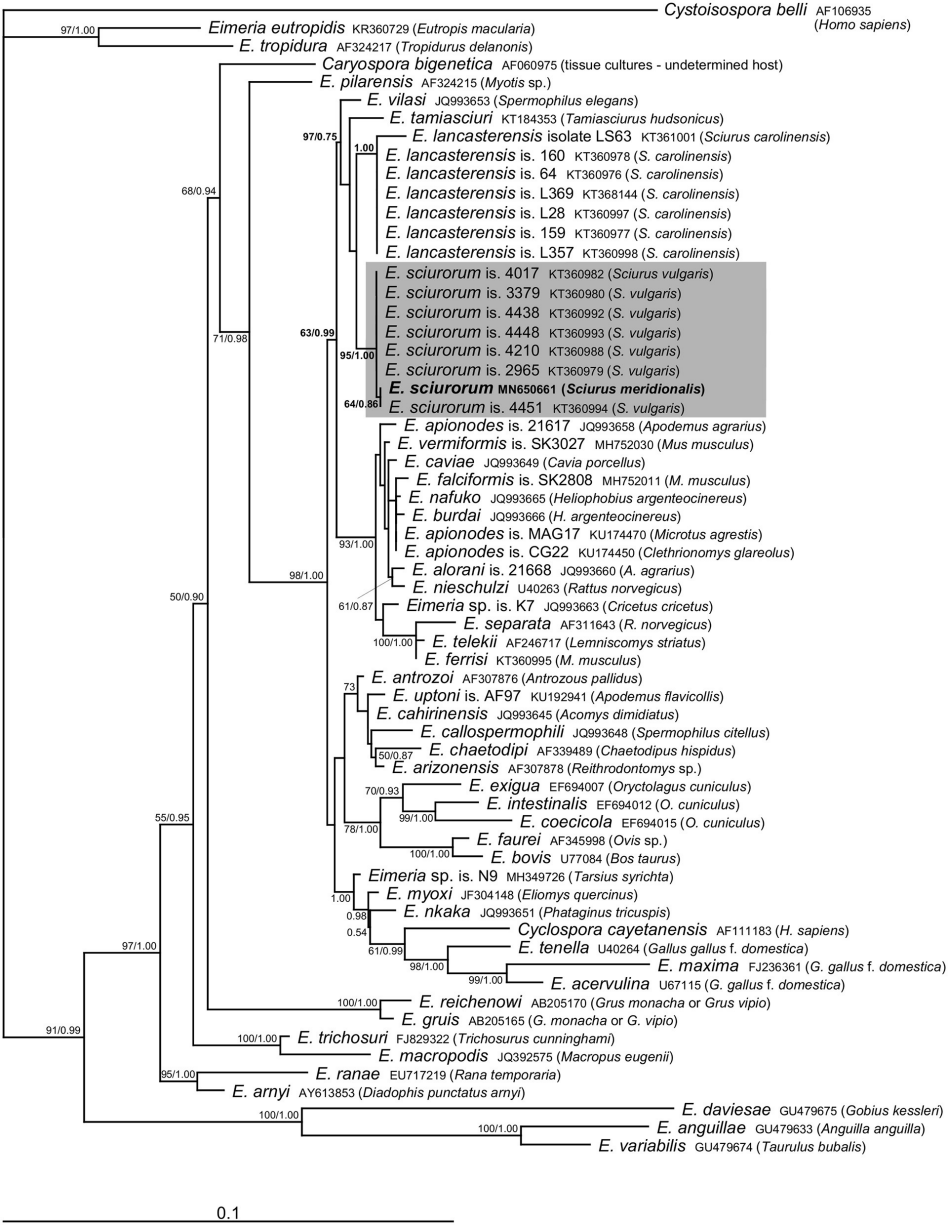
Phylogenetic relationships inferred by the ML analysis of the 18S rDNA sequences. Numbers at the nodes show bootstrap values derived from ML analysis/posterior probabilities under the BI analysis. Bootstrap supports and posterior probabilities lower than 50% or 0.50, respectively, are not provided. The scale bar represents sequence divergence. *Cystoisospora belli* is used as an outgroup.

**Figure 3:**
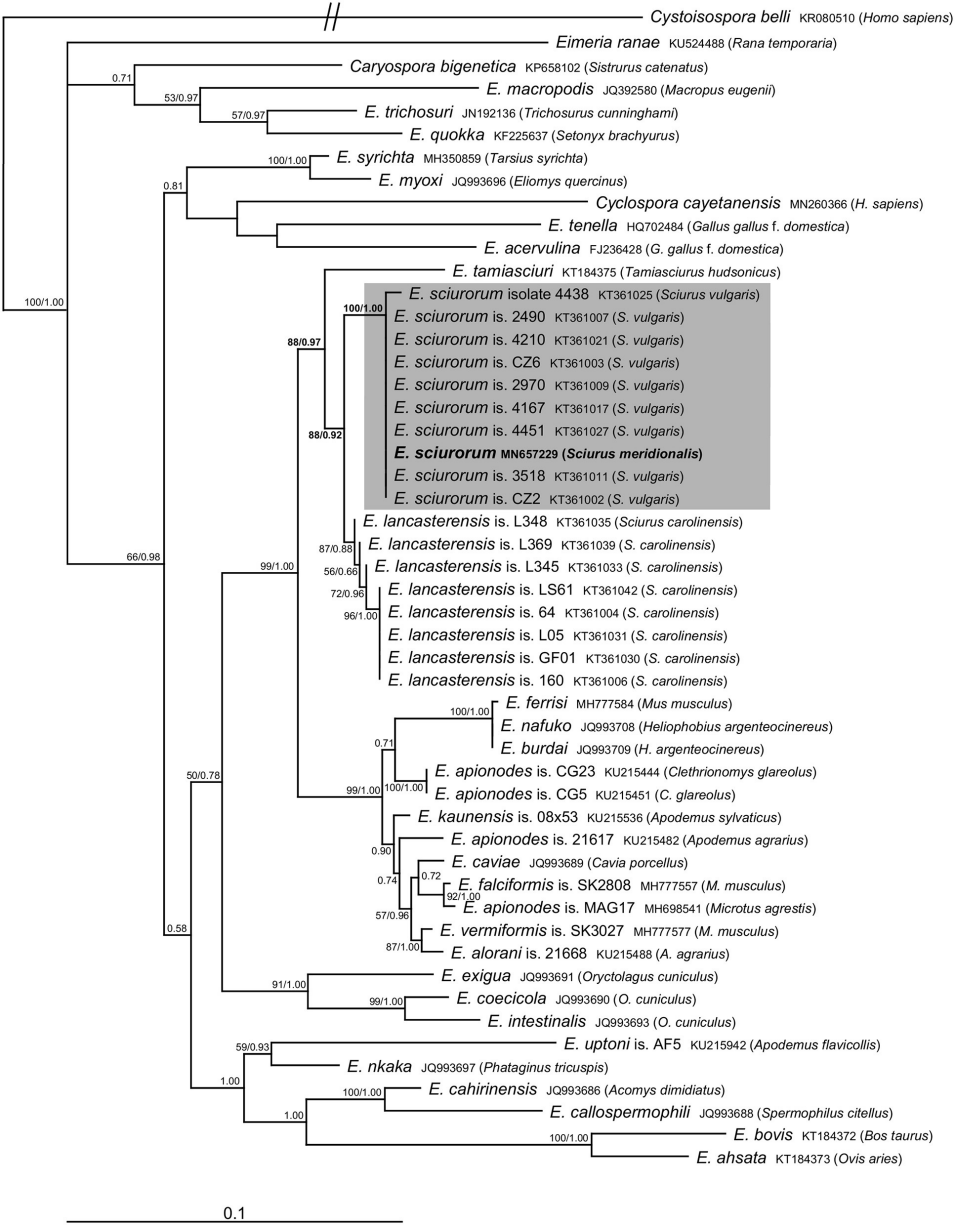
Phylogenetic relationships inferred by the ML analysis of the COI sequences. Numbers at the nodes show bootstrap values derived from ML analysis/posterior probabilities under the BI analysis. Bootstrap supports and posterior probabilities lower than 50% or 0.50, respectively, are not provided. The scale bar represents sequence divergence. *Cystoisospora belli* is used as an outgroup.

Molecular analyses of the oocysts from the ethanol-fixed fecal sample of the Calabrian black squirrel revealed the presence of sequences of 18S rRNA (1,394 bp) as well as COI (771 bp) genes belonging to the genus *Eimeria*. We did not observe mixed signal in any of the raw sequential data, which suggests that only a single *Eimeria* species was present in the sample. Comparison with sequences of *Eimeria* species available in the GenBank database suggested its affiliation to *E. sciurorum*, which was further supported by phylogenetic analyses (Figures 2, 3). Pairwise comparison based on the 18S rRNA gene also showed that *Eimeria* from *S. meridionalis* was identical with that of *E. sciurorum* isolate 4451 from *S. vulgaris* from Italy (Supplementary Table 1), while based on the COI gene, it was identical with all but one isolate of *E. sciurorum* from *S. vulgaris* from Italy and Czech Republic (Supplementary Table 2); distances of the closely related species, *E. lancasterensis*, were substantially larger (Supplementary Tables 1, 2). Molecular comparison with *E. sciurorum* described from other squirrel species than *S. vulgaris* (see the section Discussion) was not possible because these data were not available in the GenBank database.

## Discussion

The traditional species concept and the identification of eimeriid coccidia rely on the morphological features of the oocysts (size, shape, wall, internal structures), combined with data on sporulation time, endogenous development, and host specificity (2, 24, 25). However, the species identification of *Eimeria* may be further complicated by the fact that several species can co-occur in a single host individual (2, 12, 26). To reach the required resolution for distinguishing the unsporulated coccidia in our study, we combined the morphological identification with molecular taxonomy. Microscopic determination revealed only unsporulated oocysts of a single morphotype that were indistinguishable from *E. sciurorum* and/or *E. lancasterensis* based on the oocyst size and morphology of the oocyst wall (27). Subsequent molecular analyses clearly placed it into *E. sciurorum* (Figures 2, 3; Supplementary Tables 1, 2).

*Eimeria sciurorum* has been so far described in four squirrel species—*Sciurus aureogaster hypopyrrhus, Sciurus carolinensis, Sciurus niger*, and *S. vulgaris* (2, 12, 26, 28–31), and its current finding in *S. meridionalis* thus represents a new host record. In the Eurasian red squirrel (*S. vulgaris*), a native squirrel species occurring in northern Palaearct including northern Italy, it was the most often recorded coccidian species in the Italian Alps (found in 111 of 143 examined samples) (12). Until recently, *S. meridionalis* was considered a subspecies of *S. vulgaris*. However, the genetic (analyses of three mitochondrial markers), morphological (color, length, weigh, skull differences), and geographical data (disjunct distribution with respect to *S. vulgaris*) strongly supported its designation as a new species, endemic to southern Italy (32). Because the present results are based on a single individual host, no conclusions can be drawn about how common/how prevalent *E. sciurorum* may be in the Calabrian black squirrels.

Five squirrel species occur in Italy: the only native *S. vulgaris*, and the introduced (or escaped from captivity) *S. carolinensis, Callosciurus erythraeus, C. finlaysonii, and Tamias sibiricus* (27, 33, 34). The geographical range of *S. meridionalis* does not overlap with that of *S. vulgaris, S. carolinensis*, or other squirrels from which *E. sciurorum* has been recorded so far (32, 33, 35). Thus, we can exclude that this coccidium was only a random passage through the gastrointestinal tract of *S. meridionalis*. It would be optimal to carry out a cross-transmission experiment and to determine *S. meridionalis*' endogenous development; however, this is not possible in endemic wild-living animals because of their conservation status and the fact that it is difficult to obtain coccidia-free individuals.

Therefore, it is not clear how *S. meridionalis* acquired *E. sciurorum*. However, we suggest the following possible scenario. *Sciurus meridionalis* occurs only in southern Italy (Calabria and Basilicata regions), and historically, it has never geographically overlapped with the type species of *E. sciurorum, S. vulgaris*, which occurs only in northern Italy. Nevertheless, several individuals of *C. finlaysonii*, first introduced in northwestern Italy in 1981 followed by rapid expansion, were released also in Maratea (Basilicata region) in 1985. Maratea is located about 100 km north of the locality where we found the *S. meridionalis* carcass. Since then, the new introduced population of *C. finlaysonii* rapidly increased and spread from Maratea to the north (to the Campania region) (33). However, it is plausible to think that more recently few individuals moved also further to the south. Rather, a fast spread of the introduced alien squirrel species was described, e.g., by Di Febbraro et al. (34). Thus, it is possible that *C. finlaysonii* can also harbor *E. sciurorum* and could transmit this parasite to *S. meridionalis*. It has been documented that some rodent *Eimeria* species are able to exploit new hosts that live syntopically. For example, eimerians infecting *Apodemus* mice can infect also *Clethrionomys* and *Microtus* rodents (14). Exploration of the current range and parasitofauna of wild populations of *C. finlaysonii* is necessary to confirm this hypothesis.

The results of phylogenetic analyses supported Nadler's theory (6) that multihost parasites display lower genetic diversity. This theory is a potential explanation for the genetic similarity between *E. sciurorum* sequences from *S. meridionalis* and *S. vulgaris*. Except for one sequence differing in three nucleotides, the COI sequences of *E. sciurorum* from *S. meridionalis* and from several individuals of *S. vulgaris* originating from Czech Republic and Italy were identical (Figure 3, Supplementary Table 2).

The fact that at necropsy there were no remarkable gross findings other than those associated with the road traffic accident may suggest that *E. sciurorum* was not pathogenic for the present individual. So far, there are no publications focused on the pathogenicity of *E. sciurorum*. However, Simpson et al. (36) in a study on causes of mortality in red squirrels in Great Britain frequently observed the presence of small to moderate numbers of *Eimeria* sp. oocysts without any associated pathology. This may mean that squirrels are rather resistant because they got adapted to the eimerian parasites during the evolutionary process.

Parasites of the genus *Eimeria* are generally supposed to be highly host-specific. However, a few studies have provided evidence for the sharing of some species among different hosts (including squirrels). *Eimeria confusa, E. lancasterensis*, and *E. ontarioensis*, all originally described from the Eastern gray squirrel (*S. carolinensis*), are also able to infect other North American squirrel species such as *S. niger* or *S. aberti* (30, 31, 37). Hofmannová et al. (27) described the successful colonization of the European territory by *E. lancasterensis* along with its host; however, it was unable to cross the species barrier between its natural host (*S. carolinensis*) and native red squirrels (*S. vulgaris*). Finally, although we undoubtedly found *E. sciurorum* in the Calabrian black squirrel, the question remains whether it is the only coccidian species able to infect this squirrel species. Further studies focused on coprological survey of the populations of the Calabrian black squirrel would help to elucidate this question.

Based on the aforementioned example of squirrels, we would like to show the necessity of further studies on host–parasite relations and their advancement. Growing human population, industrial and agricultural development, and climate change result in a loss of natural habitats and their endemic faunas. Since coccidia are parasites of health and economic impact, each piece of information on the new host record can help us to understand the host–parasite coevolution in its complexity. Host specificity, affecting the parasite diversification, thus represents the key factor determining the spread of parasitic diseases, and having impact on disease epidemiology, ecology, and parasite evolution.

## Data Availability Statement

The sequences generated in this study are deposited in the NCBI GenBank database under the accession numbers MN650661 (18S rDNA) and MN657229 (COI).

## Ethics Statement

This study did not require a specific permit or ethical approval because the squirrel was a roadkill specimen collected opportunistically. Procedures for this study were performed in accordance with the guide for the care and use of animals by the Italian Ministry of Health.

## Author Contributions

JK analyzed the sequential data, performed the phylogenetic analyses, and wrote the manuscript. LH extracted the DNA, performed the PCR, obtained the sequences, and participated in writing the manuscript. FS and MS collected the sample, examined it coproscopically, performed the morphological analysis, and participated in writing the manuscript. All authors contributed to the article and approved the submitted version.

## Conflict of Interest

The authors declare that the research was conducted in the absence of any commercial or financial relationships that could be construed as a potential conflict of interest.

## References

[1] Johnson KP Williams BL Drown DM Adams RJ Clayton DH. The population genetics of host specificity: genetic differentiation in dove lice (Insecta: Phthiraptera). Mol Ecol. (2002) 11:25–38. 10.1046/j.0962-1083.2001.01412.x 11903902

[2] Pellérdy LP Coccidia and Coccidiosis 2nd ed Budapest: Akadémiai Kiadó, Publishing House of the Hungarian Academy of Sciences (1974).

[3] Poulin R Evolutionary Ecology of Parasites. New Jersey, NJ: Princeton University Press (2007).

[4] Poulin R Krasnov BR Mouillot D. Host specificity in phylogenetic and geographic space. Trends Parasitol. (2011) 27:355–61. 10.1016/j.pt.2011.05.003 21680245

[5] Falk BG Perkins SL. Host specificity shapes population structure of pinworm parasites in Caribbean reptiles. Mol Ecol. (2013) 22:4576–90. 10.1111/mec.12410 23848187

[6] Nadler SA. Microevolution and the genetic structure of parasite populations. J Parasitol. (1995) 81:395–403. 10.2307/3283821 7776124

[7] Archie EA Ezenwa VO. Population genetic structure and history of a generalist parasite infecting multiple sympatric host species. Int J Parasitol. (2011) 41:89–98. 10.1016/j.ijpara.2010.07.014 20828576

[8] Barrett LG Thrall PH Burdon JJ Linde CC. Life history determines genetic structure and evolutionary potential of host-parasite interactions. Trends Ecol Evol. (2008) 23:678–85. 10.1016/j.tree.2008.06.017 18947899 PMC2653456

[9] Tompkins DM Clayton DH Host resources govern the specificity of swiflet lice: size matters. J Anim Ecol. (1999) 68:489–500. 10.1046/j.1365-2656.1999.00297.x

[10] Joyner LP Host and site specificity. In: Long PL, editors. The biology of the Coccidia, Baltimore, MD: University Park Press (1982). p. 35–62.

[11] Marquardt WC. Host and site specificity in the coccidian. In: Hammond DM, Long PL, editors. The Coccidia: Eimeria, Isospora, Toxoplasma, and Related Genera. Baltimore, MD: University Park Press (1973). p. 23–42.

[12] Bertolino S Wauters LA De Bruyn L Canestri-Trotti G. Prevalence of coccidia parasites (Protozoa) in red squirrels (*Sciurus vulgaris*): effects of host phenotype and environmental factors. Oecologia. (2003) 137:286–95. 10.1007/s00442-003-1345-x 12898385

[13] de Vos AJ. Studies on the host range of *Eimeria chinchillae* de Vos & van der Westhuizen, 1968. Onderstepoort J Vet Res. (1970) 37:29–36. 5526336

[14] Mácová A Hoblíková A Hypša V Stanko M Martinu J Kvičerová J. Mysteries of host switching: diversification and host specificity in rodent-coccidia associations. Mol Phylogenet Evol. (2018) 127:179–89. 10.1016/j.ympev.2018.05.009 29753710

[15] Modrý D Petrželková KJ Kalousová B Hasegawa H Parasites of African Great Apes. Atlas of Coproscopic Diagnostics. 1st ed Brno: UVPS Brno, HPI-lab (2015).

[16] Kvičerová J Hypša V. Host-parasite incongruences in rodent *Eimeria* suggest significant role of adaptation rather than cophylogeny in maintenance of host specificity. PLoS ONE. (2013) 8:e63601. 10.1371/journal.pone.0063601 23861732 PMC3701668

[17] Hall TA BioEdit: a user-friendly biological sequence alignment editor and analysis program for Windows 95/98/NT. Nucl Acids Symp Ser. (1999) 41:95–8.

[18] Huelsenbeck JP Ronquist F. MRBAYES: bayesian inference of phylogenetic trees. Bioinformatics. (2001) 17:754–5. 10.1093/bioinformatics/17.8.754 11524383

[19] Guindon S Gascuel O A simple, fast, and accurate algorithm to estimate large phylogenesis by maximum likelihood. Syst Biol. (2003) 52:696–704. 10.1080/10635150390235520 14530136

[20] Posada D. jModelTest: phylogenetic model averaging. Mol Biol Evol. (2008) 25:1253–6. 10.1093/molbev/msn083 18397919

[21] Posada D. Selection of models of DNA evolution with jModelTest. Methods Mol Biol. (2009) 537:93–112. 10.1007/978-1-59745-251-9_5 19378141

[22] Page RDM. TREEVIEW: an application to display phylogenetic trees on personal computers. Comput Applic Biosci. (1996) 12:357–8. 10.1093/bioinformatics/12.4.357 8902363

[23] Swofford DL Phylogenetic Analysis Using Parsimony (^*^and Other Methods), Version 4. Sinauer Associates, Sunderland, Massachusetts (2001).

[24] Berto BP McIntosh D Lopes CWG. Studies on coccidian oocysts (Apicomplexa: Eucoccidiorida). Braz J Vet Parasitol. (2014) 23:1–15. 10.1590/S1984-29612014001 24728354

[25] Duszynski DW Wilber PG. A guideline for the preparation of species descriptions in the Eimeriidae. J Parasitol. (1997) 83:333–6. 10.2307/3284470 9105325

[26] Levine ND Ivens V The Coccidian Parasites (Protozoa, Sporozoa) of Rodents. Urbana, IL: Illinois Biological Monographs 33, The University of Illinois Press (1965). 10.5962/bhl.title.50242

[27] Hofmannová L Romeo C Štohanzlová L Jirsová D Mazzamuto VM Wauters LA. Diversity and host specificity of coccidia (Apicomplexa: Eimeriidae) in native and introduced squirrel species. Eur J Protistol. (2016) 56:1–14. 10.1016/j.ejop.2016.04.008 27268408

[28] Galli-Valerio B Parasitologische Untersuchungen und Beiträge zur parasitologischen Technik. Zbl Bakt Parasit, Inf Krank Hyg Abt II. (1922) 56:344–7.

[29] Lainson R Parasitological studies in British Honduras III. - Some coccidial parasites of mammals. Ann Trop Med Parasitol. (1968) 62:252–9. 10.1080/00034983.1968.11686557 4178556

[30] Motriuk-Smith D Seville RS Oliver CE Hofmann DL Smith AW. Species of *Eimeria* (Apicomplexa: Eimeriidae) from tree squirrels (*Sciurus niger*) (Rodentia: Sciuridae) and analysis of the ITS1, ITS2, and 5.8S rDNA. J Parasitol. (2009) 95:191–7. 10.1645/GE-1653.1 19245280

[31] Spurgin RJ Hnida JA *Eimeria lancasterensis* and *Eimeria ontarioensis* from fox squirrels, *Sciurus niger*, in Southeastern Nebraska, U.S.A. Comp Parasitol. (2002) 69:211–2. 10.1654/1525-2647(2002)069[0211:ELAEOF]2.0.CO;2

[32] Wauters LA Amori G Aloise G Gippoliti S Agnelli P Galimberti A New endemic mammal species for Europe: *Sciurus meridionalis* (Rodentia, Sciuridae). Hystrix It J Mamm. (2017) 28:1–8. 10.4404/hystrix-28.1-12015

[33] Aloise G Bertolino S Free-ranging population of the Finlayson's squirrel *Callosciurus finlaysonii* (Horsfield, 1824) (Rodentia, Sciuridae) in South Italy. Hystrix It J Mamm. (2005) 16:70–4. 10.4404/hystrix-16.1-4344

[34] Di Febbraro M Menchetti M Russo D Ancillotto L Aloise G Roscioni F Integrating climate and land-use change scenarios in modelling the future spread of invasive squirrels in Italy. Divers Distrib. (2018) 25:644–59. 10.1111/ddi.12890

[35] Wilson DE Reeder DM Mammal Species of the World: A Taxonomic and Geographic Reference 3^rd^ edition. Baltimore, MD: The Johns Hopkins University Press (2005).

[36] Simpson VR Hargreaves J Butler HM Davison NJ Everest DJ. Causes of mortality and pathological lesions observed post-mortem in red squirrels (*Sciurus vulgaris*) in Great Britain. BMC Vet Res. (2013) 9:229. 10.1186/1746-6148-9-229 24238087 PMC4225685

[37] Joseph T. Experimental transmission of *Eimeria confusa* Joseph 1969 to the fox squirrel. J Wildl Dis. (1975) 11:402–3. 10.7589/0090-3558-11.3.402 1152180

